# Variations in Environmental Signals in Tree-Ring Indices in Trees with Different Growth Potential

**DOI:** 10.1371/journal.pone.0143918

**Published:** 2015-11-30

**Authors:** Polona Hafner, Jožica Gričar, Mitja Skudnik, Tom Levanič

**Affiliations:** 1 Department of Forest Yield and Silviculture, Slovenian Forestry Institute, Ljubljana, Slovenia; 2 Department of Forest and Landscape Planning and Monitoring, Slovenian Forestry Institute, Ljubljana, Slovenia; Wuhan Botanical Garden,CAS, CHINA

## Abstract

We analysed two groups of *Quercus robur* trees, growing at nearby plots with different micro-location condition (W-wet and D-dry) in the floodplain Krakovo forest, Slovenia. In the study we compared the growth response of two different tree groups to environmental variables, the potential signal stored in earlywood (EW) structure and the potential difference of the information stored in carbon isotope discrimination of EW and latewood (LW). For that purpose EW and LW widths and carbon isotope discrimination for the period 1970–2008 AD were measured. EW and LW widths were measured on stained microscopic slides and chronologies were standardised using the ARSTAN program. α-cellulose was extracted from pooled EW and LW samples and homogenized samples were further analysed using an elemental analyser and IRMS. We discovered that W oaks grew significantly better over the whole analysed period. The difference between D and W oaks was significant in all analysed variables with the exception of stable carbon isotope discrimination in latewood. In W oaks, latewood widths correlated with summer (June to August) climatic variables, while carbon isotope discrimination was more connected to River Krka flow during the summer. EW discrimination correlated with summer and autumn River Krka flow of the previous year, while latewood discrimination correlated with flow during the current year. In the case of D oaks, the environmental signal appears to be vague, probably due to less favourable growth conditions resulting in markedly reduced increments. Our study revealed important differences in responses to environmental factors between the two oak groups of different physiological conditions that are preconditioned by environmental stress. Environmental information stored in tree-ring features may vary, even within the same forest stand, and largely depends on the micro-environment. Our analysis confirmed our assumptions that separate EW and LW analysis of widths and carbon isotope discrimination provides complementary information in *Q*. *robur* dendroecology.

## Introduction

The vitality of trees is one of the most important indicators of forest condition. Several studies have clearly shown different vitality and growth patterns of trees of the same species growing at the same site [1–3]. The reasons for such a phenomenon are rarely researched in detail, although the outcome of reduced tree growth can be studied in various regards, including wood structure and quality [4], stand dynamics and assessment of tree mortality [1] and loss of ability to respond to changing environmental variables [5].

A multiproxy approach has been adopted in many studies of the relationship between tree growth and environmental variables in recent years [6, 7]. Among many methods used, stable isotopes appear to be a particularly valuable tool when studying climate-growth relationships of trees in temperate climatic regions [8]. Namely, tree growth is influenced by a complex combination of environmental variables, resulting in a lack of a strong climatic signal in tree-ring widths [9–11] at sites near to ecological limits, where tree growth is usually controlled by a low number of important environmental variables [12]. The ratio of stable carbon isotopes in leaf tissue is a result of fractionation during CO_2_ diffusion through the stomata and carboxylation. Both processes are influenced by plant physiological and environmental conditions [13]. Where irradiance and temperature are the limiting factors, the dominant control of stable carbon isotopic composition (δ^13^C) may be the photosynthetic rate. On the other hand, stomatal conductance dominates in moisture-stressed environments and δ^13^C correlates strongly with moisture parameters [14]. Simultaneous analysis of several tree-ring variables increases the strength of climate correlations and extends the range of extractable parameters [6]. Studying several tree-ring variables should also be helpful in detecting the most important environmental variable from tree-rings and interpreting the differences [15]. It has been shown that tree-ring widths (TRW) and stable carbon isotope composition (δ^13^C) have different temporal patterns [16, 17].

Due to the longevity of oaks and their durable wood, tree rings are an important proxy in dendrochronological studies [18]. [19]. In oaks, tree-ring and latewood widths (LW-W) have been mostly used in dendroclimatological studies, while earlywood widths (EW-W) are usually ignored. So far, only a few studies have included stable carbon isotopes of *Q*. *robur* tree-rings for Southeast Europe. Kern, Patkó [20] found a strong correlation between late wood (LW) widths, δ^13^C in LW and June precipitation. A comparative study of surviving and dead *Q*. *robur* trees showed significant differences among tree-ring variables, including carbon isotope discrimination (Δ) [21]. For early wood (EW), it was shown that width [22, 23] and δ^13^C [9] contain weaker or even no climatic signal compared to LW. However, the anatomical structure of EW has proven to be a promising environmental proxy [24] and shows great potential for better understanding the biochemical processes of carbon isotopes incorporation within the tree [25]. In our recent study, it was shown that environmental information in wood-anatomical variables of flooded and non-flooded pedunculated oaks may vary within the same forest stand [26].

In this study, we examined the response to the environmental conditions in an oak population. We assumed that there are differences in the response due to different growth rates. For this purpose, we examined the potential of tree-ring growth indices and Δ in *Q*. *robur* for dendroclimatological and dendroecological analysis. We selected two groups of *Q*. *robur* trees with different growth patterns growing in a floodplain forest in Slovenia. We anticipated that information on the environmental sensitivity of EW would be of particular value in ring-porous trees with narrow tree-rings containing a negligible proportion of LW. We also checked whether environmental information stored in Δ of EW and LW of oaks with different growth potential is additional or redundant information.

## Material and Methods

### Study site characteristics

**Fig 1:**
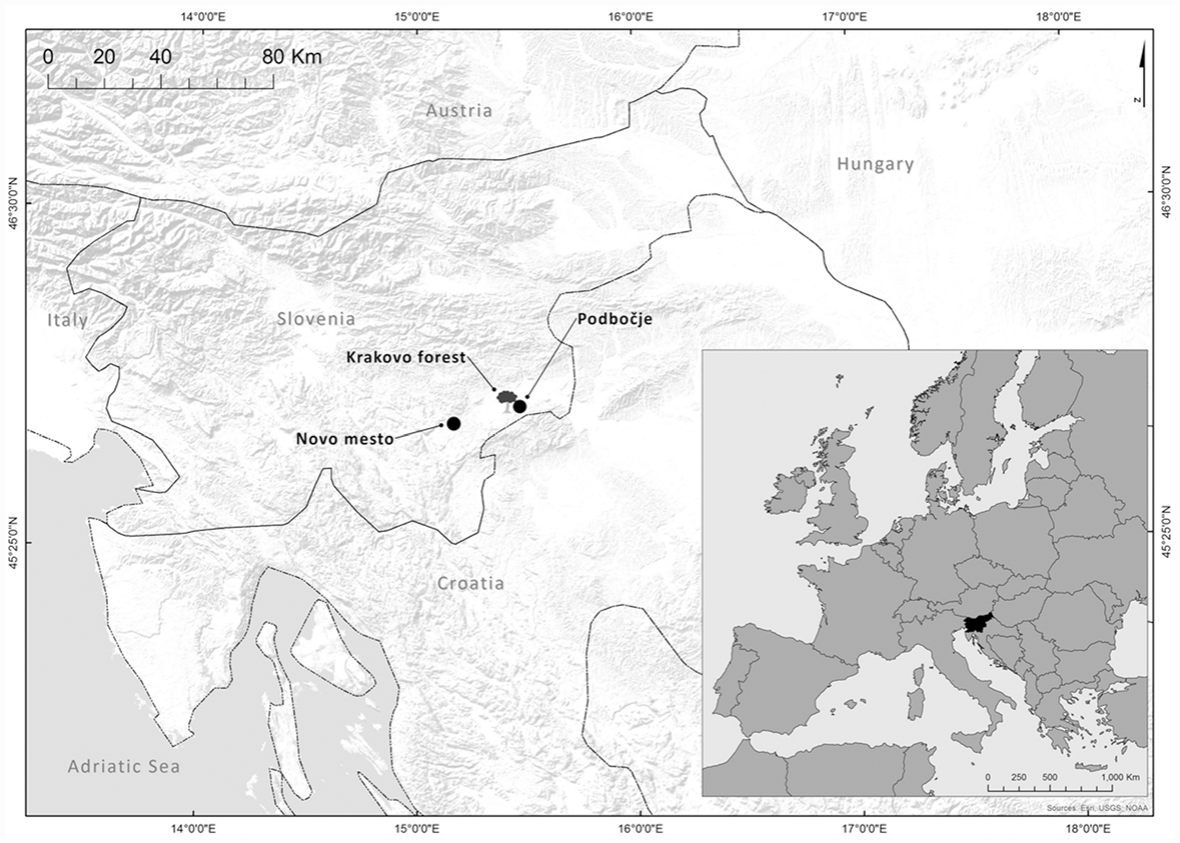
Map of study area. Marked are study site (Krakovo forest), meteorological station (Novo mesto) and river measurement station (Podbočje). Map used: World Terrain Base; data sources: Esri, USGS, NOAA); Reprinted from PLOS ONE under a CC BY license, with permission from ESRI Licence Agreement E204 06/13/2014, original copyright June 2009.

The study site was located in Krakovo *Querco robori*–*Carpinetum* forest (45°54’N, 15°25’E, elevation 150 m), the largest floodplain lowland oak forest complex in Slovenia (Fig 1). It is an even-aged forest, where dominant and co-dominant trees prevail. Permission for the field sampling was granted by Slovenia Forest Service. The field study did not involve endangered or protected species.

**Fig 2:**
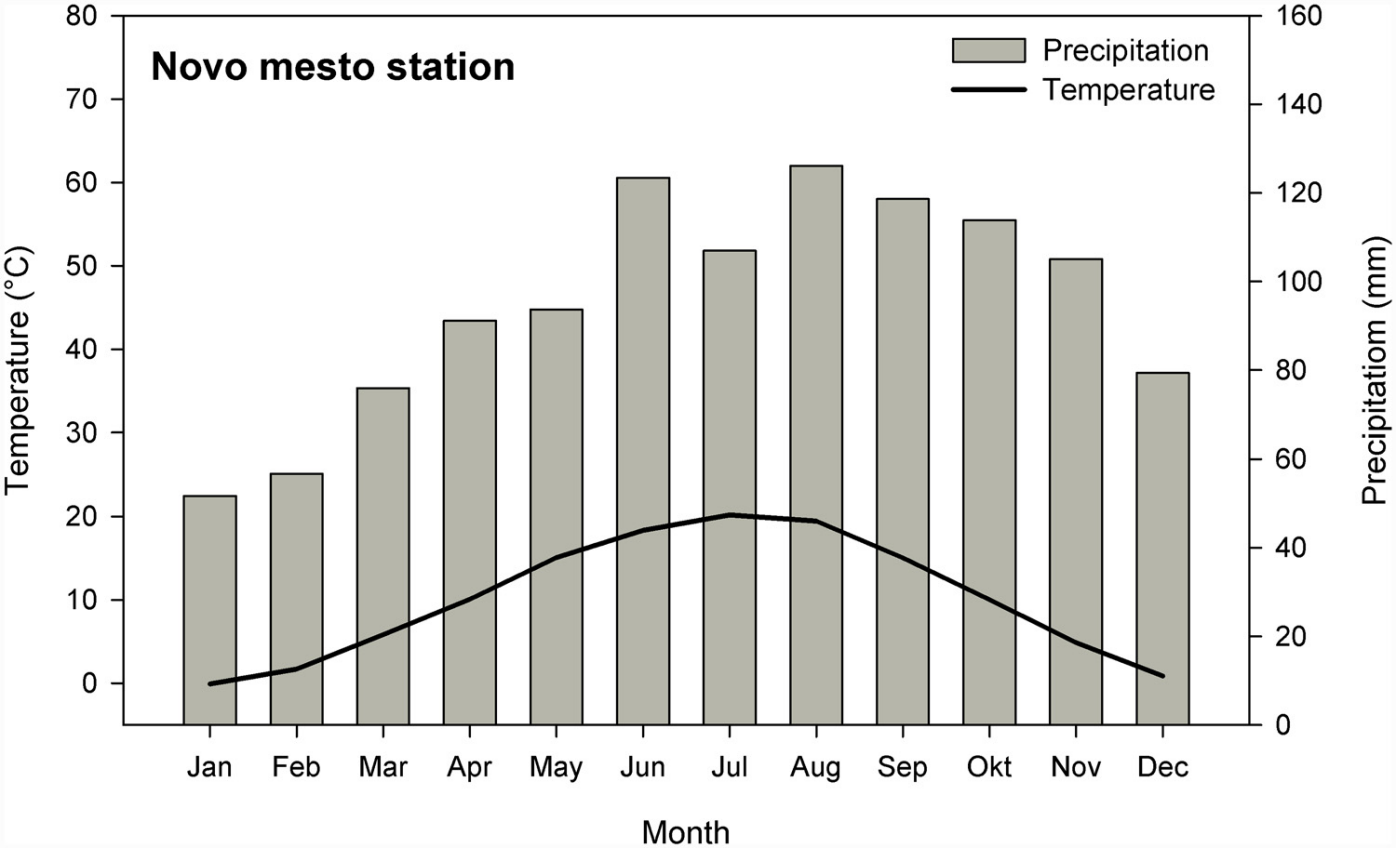
Climatic diagram for Novo mesto meteorological station for the period 1970–2008. Mean monthly temperatures and monthly sum of precipitation are denoted by lines and bars, respectively.

The site is influenced by the sub-pannonian continental climate. The mean summer temperature in the period 1970–2008 measured at the nearby Novo mesto weather station is 19.3°C and the average winter temperature is just below zero (–0.8°C). Total annual precipitation is 1,149 mm, with the majority (70%) falling during the growing season (March–October) (Fig 2). Meteorological data for the Novo Mesto station were obtained from the Slovenian Environment Agency.

At the study site, hydromorphic soils, pseudogley and amphygley, with low infiltration capacity prevail and, together with the microtopography, they greatly influence the runoff, distribution and water movement in the soil. The groundwater level depends on the arrangement of different forms of deposits and differences in permeability of the soil surface. In rainy periods, some parts of the forest can remain flooded for several weeks and water slowly evaporates or is absorbed by the soil. Due to the low infiltration capacity of this soil type and in the case of a slightly slope, most of the water on slopes can runoff before absorption, which can have a negative influence on the micro-site hydrological conditions [27].

**Fig 3:**
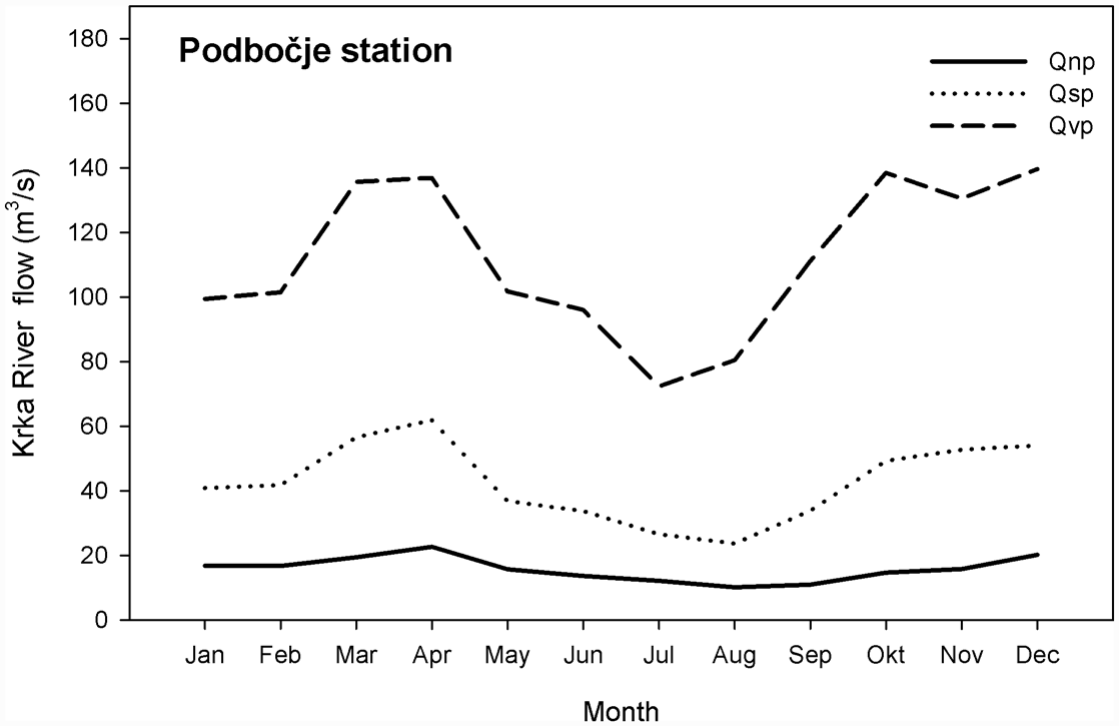
Average monthly flow of the Krka River for the period 1970–2008. Data are minimum (Qnp), average (Qs) and maximum (Qvp) monthly rates of flow (daily average) in m^3^/s.

In addition to rainwater and groundwater levels, the Krka River also affects the hydrological conditions in Krakovo Forest. It is a Karst river and has a rain-snow regime, with runoff peaks in April and November, and minima in August and January (Fig 3). Data for minimum (Qnp), average (Qsp) and maximum (Qvp) monthly rate of River Krka flow for the Podbočje station were obtained from the Slovenian Environment Agency, for the period 1970–2008.

### Tree selection and construction of tree-ring width chronologies

Penduculate oak (*Q*. *robur* L.) trees were sampled at two research plots. Plots are about 600 m apart and located in the same forest association, but they differ in their hydrological conditions. The first plot is occasionally flooded (W—wet oaks), whereas the second plot (D—dry oaks) remains dry throughout the growing season. To exclude the effect of stand to the minimum, six (co)dominant trees were selected and sampled at each plot with the approval of the Slovenia Forest Service. Selected trees were 80–100 yeas old, with DBH 30–60 cm, without any visible mechanical injuries on stems or roots.

During winter 2008–2009, we took 10 mm wide cores about 1.3 m above the ground from each of the trees in order to prepare microscopic slides. Each core was cut exactly at the growth ring boundary into pieces about 5–6 cm long, so that they could be placed on microscope slides. Permanent transverse sections of 25 μm in thickness were prepared on a “G.S.L. 1” Sledge microtome (Gärtner and Schweingruber; Design and production: Lucchinetti, Schenkung Dapples, Zürich, Switzerland) with disposable blades. Sections were stained with safranin (Merck, Darmstadt, Germany) (0.5% in 95% of ethanol) and mounted in Euparal and observed under an Olympus BX51 (Tokyo, Japan) light microscope and analysed with the Nikon NIS-Elements Basic Research v.2.3 image analysis system (Tokyo, Japan).

We measured the widths of tree ring, earlywood (EW) and latewood (LW) of the last 39 rings (1970–2008) in order to avoid juvenile wood, because its anatomical structure and tree-rings significantly differ from adult wood [4, 28]. Individual raw measurements were then detrended using ARSTAN software [29] by applying a two-step procedure. Long-term and growth trends were removed using a negative exponential function. This procedure was followed by a 67% cubic smoothing spline with a 50% cut-off frequency to remove any effect of stand competition and disturbance events [30]. The autocorrelation was removed by applying an autocorrelation filter to the detrended measurements. Indexed chronologies were combined using bi-weight robust estimation of the mean to obtain residual chronologies. In statistical analysis, averaged raw EW-W (earlywood width) and LW-W (latewood width) chronologies were used for the calculation of Student’s test statistics, while detrended EW-W and LW-W chronologies were used for calculation of Pearson’s coefficients.

### Isotope measurement

For stable isotope analysis, cross-dated tree-rings were divided into EW and LW portions with a scalpel under an Olympus SZ-60 binocular microscope (Tokyo, Japan). Samples of EW and LW were separately pooled to create two annualized records. Altogether 78 samples were measured, 39 samples for EW and 39 for LW, each in two replicates per sample to ensure reliable results and to verify analytical precision. The advantage of pooling is in reducing the time of sample preparation and costs. Previous studies have shown very close δ^13^C values of pooled samples and average δ^13^C values of individually analysed same trees [31]. α-cellulose was extracted from the samples by the following standard techniques [32, 33], homogenized using a Hielscher ultrasonic probe and freeze-dried for 48 hours. Two replicates of 300–350 μg of dry α-cellulose were weighed into tin capsules. Samples were combusted using a PDZ Europa ANCA GSL elemental analyser and CO_2_ gas was introduced online into a PDZ Europa 20–20 stable isotope ratio mass spectrometer. Results are expressed using standard delta notation (δ^13^C) in per mille (‰) relative to the Vienna Pee Dee Belemnite (VPDB) standard. The precision of analysis of the laboratory standard was 0.08‰ (n = 64 internal standards).

Stable carbon isotope values are expressed as isotope discrimination (Δ). By expressing the ^13^C/^12^C ratio in terms of discrimination of carbon isotopes by C3 plants, we distinguish variations in atmospheric δ^13^CO_2_ due to fossil burning from the effect of plant metabolic processes. Discrimination is expressed as [34]:

$$\Delta \;=\;\left(\delta^{13}C_{air}\;-\;\delta^{13}C_{sample}\right)\;/\;\left(1\;+\;{(\delta }^{13}C_{sample}\;/\;1000)\right)$$ (1)

where *δ* ^*13*^ *C*_*air*_ and *δ* ^*13*^ *C*_*sample*_ represent the isotopic composition of atmosphere and tree-ring samples, respectively.

Pearson’s correlation coefficients were calculated and t-tests were done using the program, R version 2.8.0. [35]. All graphs were produced using SigmaPlot (version 11) software.

Map in Fig 1 was created using ArcGIS software by Esri [36]. ArcGIS and ArcMap are the intellectual property of Esri and are used herein under license. Copyright Esri. All rights reserved. For more information about Esri software, please visit www.esri.com.

## Results

### Tree-ring parameters of D and W oaks

**Fig 4:**
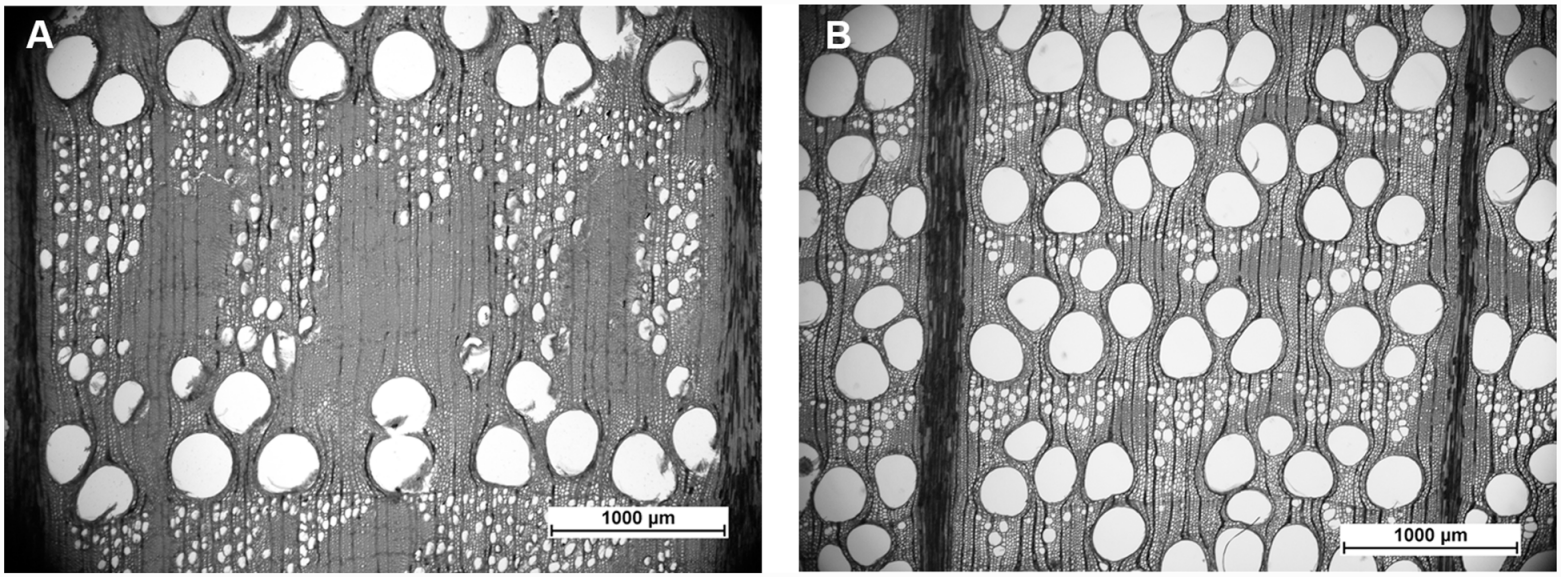
Tree-ring structure of W and D oak. (A) Wide tree-ring of W oak with a large amount of LW. (B) Narrow tree-ring of D oak containing only small proportion of LW.

**Fig 5:**
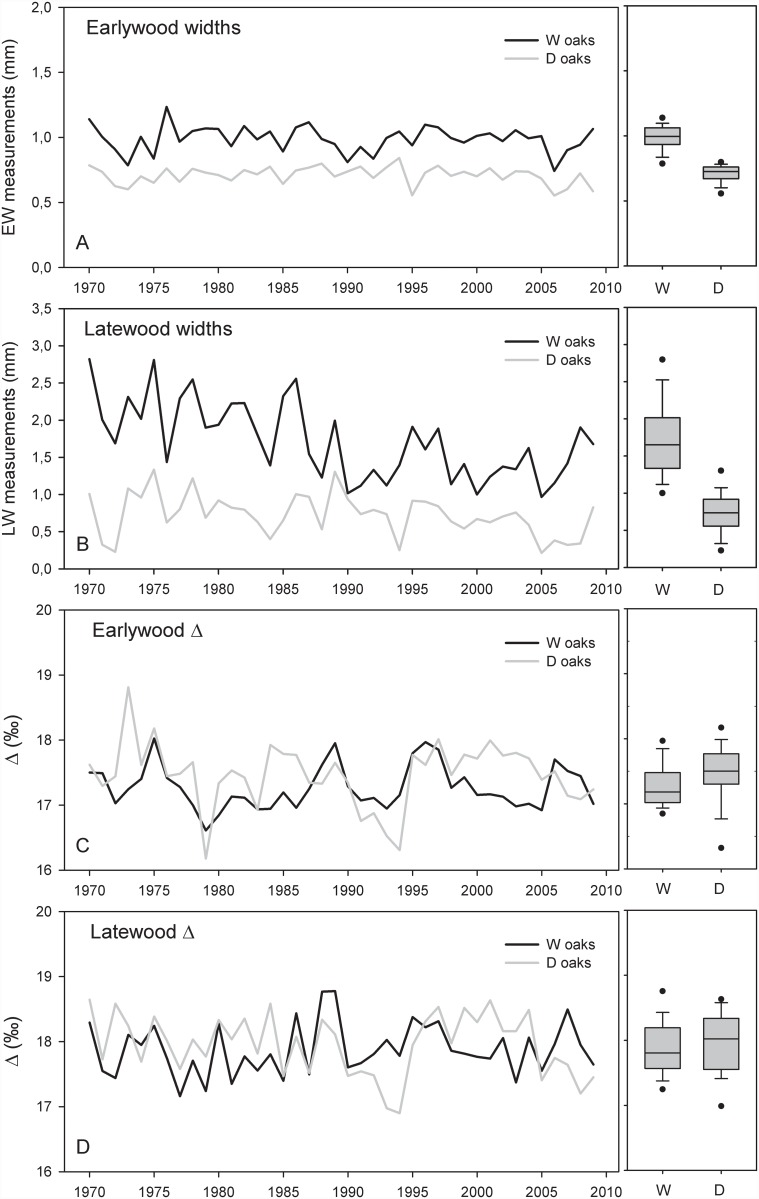
Time series of anlysed tree-ring parameters. (A) Raw earlywood widths, (B) raw latewood widths, (C) earlywood carbon isotope discrimination and (D) latewood carbon isotope discrimination of wet (W) and dry (D) oaks for the period 1970–2008.

Tree-ring widths of oaks from the wetter plot (W oaks) were considerably wider than those of oaks from the drier plot (D oaks) over the whole analysed period (i.e., 1970–2008) (Fig 4). Average widths of EW and LW significantly differed between D and W oaks; t = 15.195, p < 0.001 and t = 16.210, p < 0.001, respectively (Table 1). The difference in growth trends became particularly visible around 1990. A tree-ring width decline in D oaks was apparent in LW, where widths were narrower than 0.2 mm in the last analysed years (Fig 5A) indicating negligible production of LW. EW-W in the two analysed groups of oaks were fairly constant (Fig 5B). There was a weak but significant difference in Δ in EW (t = –2.522, p < 0.05) between the oak groups (Fig 5C) but no difference in Δ in LW (t = –0.925, p > 0.10) (Fig 5D).

**Table 1:** Descriptive statistics of analysed tree-ring parameters for W and D oaks.

	W oaks				D oaks			
	Avg.	CV (%)	Min	Max	Avg.	CV (%)	Min	Max
EW-W (mm)	0.95	14.0	0.60	1.35	0.58	15.5	0.33	0.76
LW-W (mm)	1.66	30.8	0.89	3.11	0.53	51.3	0.05	1.18
EW-Δ (‰)	17.27	1.9	16.61	18.02	17.47	2.8	16.18	18.81
LW-Δ (‰)	17.88	2.2	17.16	18.78	17.95	2.6	16.90	18.65

Presentation of average value (Avg.), coefficient of variation (CV), minimum (Min) and maximum (Max) values of earlywood widths (EW-W), latewood widths (LW-W), earlywood carbon isotope discrimination (EW-Δ) and latewood carbon isotope discrimination (LW-Δ). Average value, minimum and maximum values are presented in millimetres [mm], coefficient of variation is presented in percent [%].

In W oaks, a significant correlation was observed between LW parameters of the previous year and EW parameters of the current year. In both groups, highly statistically significant correlation existed between the current year’s Δ of EW and Δ of LW and the same is true for widths (Table 2).

**Table 2:** Correlation in tree-ring widths and carbon isotope discrimination.

	W oaks		D oaks	
	EW-W_(t)_	EW-Δ_(t)_	EW-W_(t)_	EW-Δ_(t)_
LW-W_(t-1)_	0.45	-	0.55	-
LW-W_(t)_	0.15	-	-0.07	-
LW-Δ_(t-1)_	-	0.45	-	0.37
LW-Δ_(t)_	-	0.57	-	0.65

Correlation between earlywood and latewood widths (LW-W) and carbon isotope discrimination (LW-Δ) of the previous (t-1) and current (t) year.

### The relationship between tree-ring parameters, climate and River Krka flow

**Fig 6:**
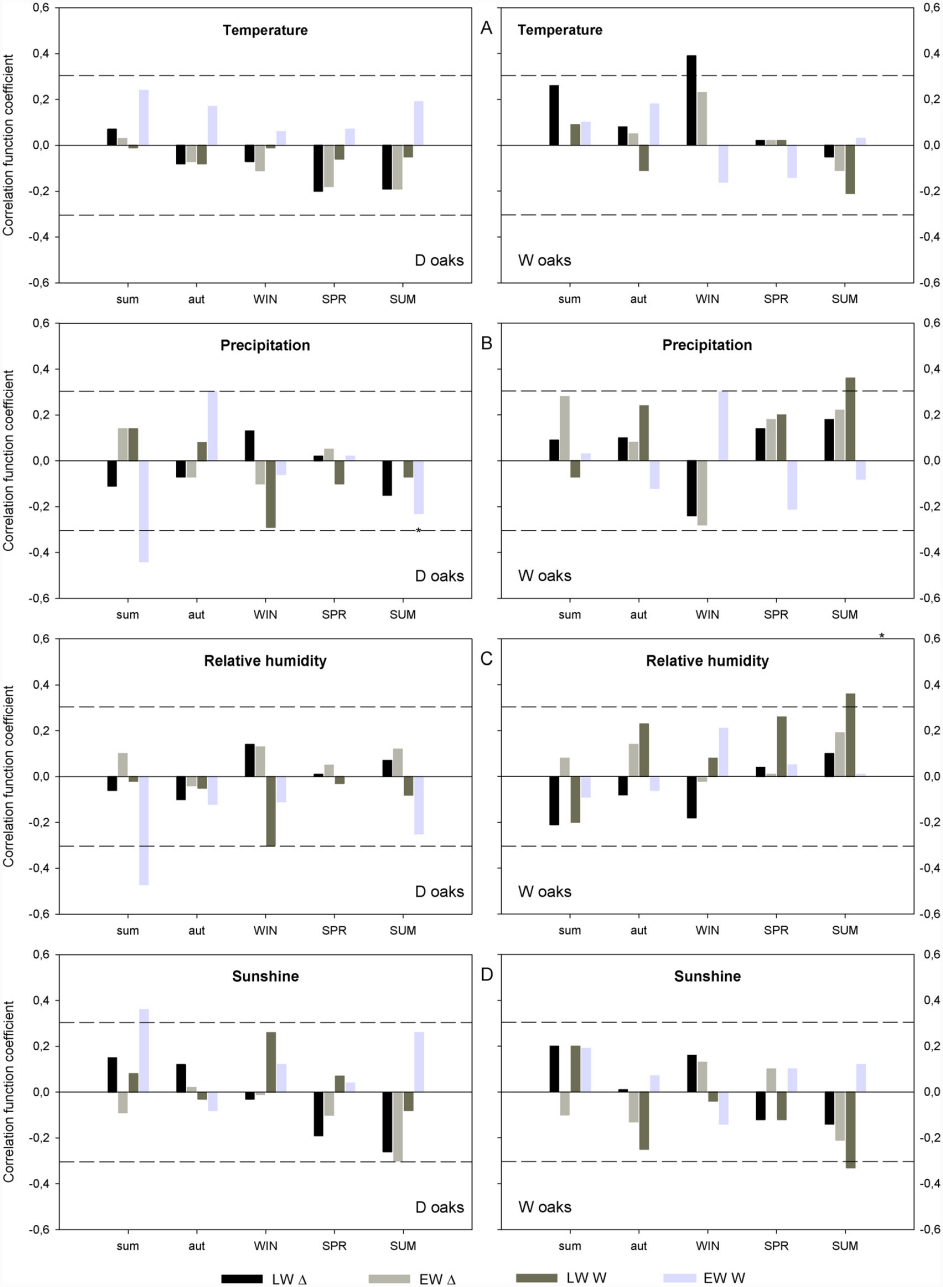
Correlation between analysed tree-ring parameters and meteorological variables. Correlation with **A** temperature, **B** precipitation, **C** relative humidity and **D** sunshine on a seasonal time scale (from previous to current summer). The dashed line denotes statistically significant values at a level of 95%. EW-Δ–earlywood discrimination, LW-Δ–latewood discrimination, EW-W—earlywood width, LW-W—latewood width, SUM—summer (from June to August), AUT—autumn (from September to November), WIN—winter (from December to February), SPR—spring (from March to May). Small letters denote the previous year.

Temperature did not affect EW and LW features of D oaks (Fig 6A). EW-W of D oaks responded negatively to a wet (Fig 6B) and humid (Fig 6C) summer of the previous year, while a sunny summer (Fig 6D) and wet autumn (Fig 6B) of the previous year promoted its width. With EW-Δ of D oaks, current summer is negatively correlated with summer sunshine duration (Fig 6D).

In the case of W oaks, a positive correlation between LW-Δ and a warm winter was apparent (Fig 6A). LW-W of W oaks was promoted by rainy (Fig 6B), humid (Fig 6C) and cloudy (Fig 6D) conditions during the current year’s summer.

**Fig 7:**
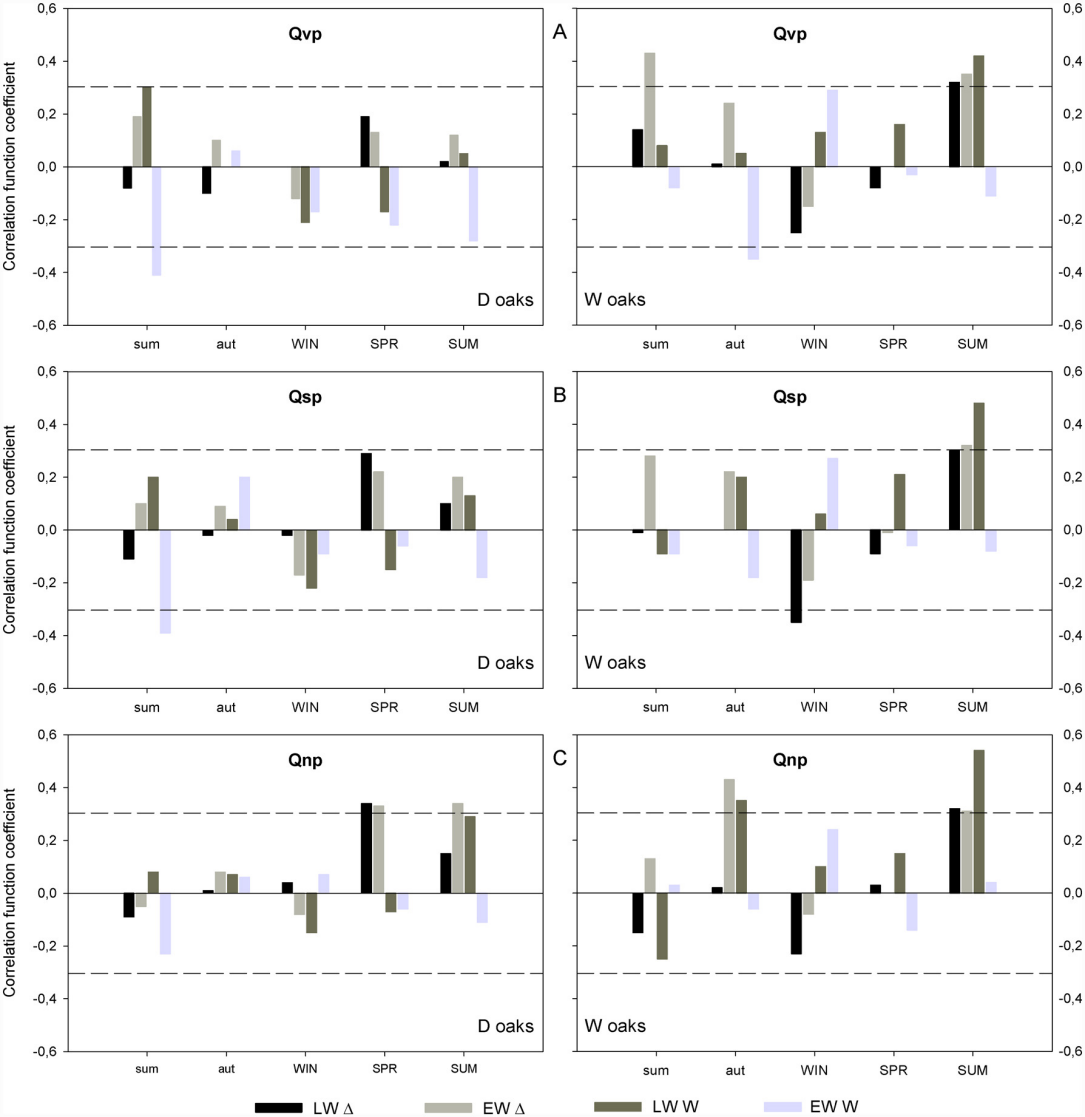
Correlation between analysed tree-ring parameters and the Krka River flow. Correlation with **A** maximum, **B** minimum and **C** mean flow on a seasonal time scale. The dashed line denotes statistically significant values at a level of 95%. EW-Δ–earlywood discrimination, LW-Δ–latewood discrimination, EW-W—earlywood width, LW-W—latewood width, SUM—summer (from June to August), AUT—autumn (from September to November), WIN—winter (from December to February), SPR—spring (from March to May). Small letters denote the previous year. Qnp—minimum flow, Qsp—mean flow, Qvp—maximum flow. Small letters denote the previous year.

In the case of D oaks, LW-W was wider when the River Krka flow of the previous summer was high (Fig 7A). In contrast, EW-W of D oaks was narrower when the River Krka flow of the previous summer was high (Fig 7A and 7B). EW-Δ was positively correlated with a high minimum flow during the spring and summer, while LW-Δ was positively correlated only with the spring minimum River Krka flow (Fig 7C). In W oaks, high River Krka flow during the summer was positively correlated with EW-Δ, LW-Δ and LW-W (Fig 7A, 7B and 7C). EW-Δ was positively correlated with a high maximum flow in the previous summer (Fig 7A). EW-W was negatively correlated with high river flow during previous autumn (Fig 7A). LW-Δ was negatively correlated with low mean River Krka flow in the winter (Fig 7B). EW-Δ was positively correlated with a high minimum flow during the previous autumn (Fig 7C). LW-W was promoted when the previous autumn River Krka flow was high (Fig 7C).

## Discussion

### Climate and hydrological signals in tree-ring widths and Δ in *Q*. *robur*

The width and stable isotope composition of EW have rarely been used in dendroclimatological studies, since it is known that the EW of deciduous species is mainly synthesized from stored photoassimilates and does not reflect the current year’s conditions [37, 38]. Additionally, it has been shown that no or only a weak response to temperature or precipitation is apparent in EW indices [20, 39, 40]. However, our aim was to check any environmental information stored in EW, especially in trees with little or hardly any LW. Our analysis showed that potential climatic information in the EW-W of D oaks depends on the previous summer’s precipitation, relative humidity and sunshine duration, as well as on the previous autumn’s precipitation. The current year’s climatic and hydrologic conditions appeared to have little influence on analysed EW parameters of D oaks, while LW parameters (with the exception of the correlation between LW-W and winter RH) showed no response to climatic variables. The most likely explanation is that the micro-environmental conditions for radial growth of D oaks are less favourable, indicating that the trees might experience physiological stress. This results in diminished wood increments, lower proportion of LW and weak response to environmental variables. It can be speculated that in such limiting conditions, the majority of carbohydrate and nutrient sources are used for EW vessel formation in order to ensure a sufficient water supply for the tree, which is crucial for its survival, since older EW vessels usually become embolized [41]. The conductive function has priority over the mechanical as the tree stem increases in diameter because the need for additional strength becomes less crucial [4, 42]. Decreasing growth curves are among the most obvious growth-related characteristics of tree decline [1]. Tree-ring widths of W oaks, on the other hand, contain similar climatic information as oaks from nearby locations in Southeast Europe, where a high amount of precipitation and moderate temperatures during the summer appear to be the main climatic factors promoting growth of *Q*. spp. [19, 20, 43]. Studied groups of W and D oaks yield different environmental information. In general, EW parameters of D oaks contain climatic information of the previous year, while in the case of W oaks, it is stored in LW parameters.

Stable isotopic composition of tree-rings proved to be a valuable tool in dendroclimatological studies in trees at temperate sites, where their growth is influenced by a combination of environmental factors, demonstrating a strong positive correlation with average summer temperature [8, 9, 20], including sunshine hours, cloud cover and precipitation at sites near the species distribution border [10]. In the case of W oaks, the high summer precipitation and high summer River Krka flow correlated with high LW-Δ. Comparison of tree-ring widths and δ^13^C in *Q*. *robur* from drier and wetter sites in Eastern England revealed higher although not statistically significant correlation of δ^13^C with environmental variables at the drier site than at the wetter one. Additionally, δ^13^C indices from the LW showed higher correlations with environmental variables and yield considerably more environmental information than the tree-ring width alone [9]. In general, tree-ring widths and Δ of W and D oaks in our research provide information on different environmental variables for different time periods during the growing season. W and D trees do not yield the same nor even similar signals stored in analysed tree-ring variables.

In D oaks, the high minimum River Krka flow during spring was positively correlated with EW-Δ and LW-Δ values, while summer flow was correlated only with EW-Δ. It has been reported that production of EW cells in *Q*. *petraea* started at the beginning of April and ceased by the end of May [44, 45]. In the light of these findings, the positive correlation between EW-Δ of D oaks and high minimum River Krka flow, together with the negative correlation between EW discrimination and sunshine duration during the current summer, has no logical explanation. However, the high correlation between EW and LW discrimination may indicate that LW of D oaks, which seem to grow in stressed conditions, may be partly synthetized from photosynthetic assimilates produced during EW formation. It is likely that D oaks suffer from carbon starvation caused by reduced photosynthesis because of very limited carbon uptake due to stomatal closure but continued metabolic demand for carbohydrates [46]. Another possible reason for the impact of the summer climatic conditions on analysed EW characteristics would be the ongoing development of EW cells in June. This would also explain the high correlation between the summer (from June to August) River Krka flow and EW-Δ in W oaks.

### Environmental sensitivity of *Q*. *robur* trees with different growth rates

This study provides new stable carbon isotope composition data for the tree-ring database in Southeastern Europe, in which only few *Q*. spp. stable isotope chronologies exist. Levanič et al. [21] compared tree-ring widths, BAI and Δ of LW in dying and surviving pedunculate oaks. They observed significant differences in all parameters analysed between the two groups. Trees that survived exhibited a relatively constant growth increment and increased Δ values compared to dying trees. Helama et al. [2] compared healthy, declining and dead *Q*. *robur* trees and noted that healthy oaks had wider increments of EW and LW than declining or dead oaks over their entire life span. Similarly, our results showed that W oaks grew considerably better over the entire studied period compared to D oaks. LW and EW-Ws of W oaks yield a similar climatic signal as oaks in other studies [20, 43], while D oaks differ in increment widths, as well as in their response to environmental conditions. Discrimination of the carbon isotope in W oaks yields little climate information but, despite the indirect influence of the Krka flow, tree-ring variables yield a potential hydrological signal.

It is also important to stress that our research is based on *Q*. *robur* samples only, while many dendroclimatic studies combine samples of *Q*. *robur and Q*. *petraea* in the same chronology [19]. It is difficult to identify these two species based solely on their wood anatomical characteristics, whereas ring-width series can be successfully cross-dated; they are therefore often treated as one species in dendrochronological studies: *Q*. *spp*. [47]. However, studies of oak growth and its relation to environmental factors largely depend on the micro-environment [26], which is usually greatly affected by soil properties. Additionally, the microscopic structure of wood [48] and ecology of *Q*. *robur* differs from that of *Q*. *petraea*; the overall response to climate is modulated by the species ecology and climatic variables are not a coherently controlling growth response of both species [49]. On the other hand, several studies have dealt with different eco-physiological strategies and spatiotemporal tree-ring signals in Mediterranean oaks, co-occurring in the same stand, and they confirmed the expected differences among the species [50–52].

Our analysis confirmed our assumptions that separate EW and LW analysis of widths and Δ provides complementary information in understanding of *Q*. *robur* response to environmental stress. In general, Δ contains little climate information in contrast to the above mentioned studies. However, it reveals important differences in response to environment between the two groups of trees of different physiological conditions, which is preconditioned by environmental stress. Thus, environmental information stored in tree-ring features may vary, even within the same forest stand, and largely depends on the micro-environment. Additionally, the relationship between River Krka flow and tree-ring parameters shows that Δ and anatomical variables in W oaks [26] appear to be a promising proxy for hydrological studies; but trees should be carefully selected for this purpose. Nevertheless, for more comprehensive dendrohydrological studies, a larger number of trees should be sampled and the groundwater level should be regularly monitored.
